# Profound Hemolytic Anemia in Babesiosis With Lyme Disease Coinfection and Occult Strongyloidiasis

**DOI:** 10.7759/cureus.115255

**Published:** 2026-08-26

**Authors:** Nikola Jovanovic, Theresa Bent, Aladin Altic

**Affiliations:** 1 Internal Medicine, Jacobi Medical Center, New York, USA

**Keywords:** babesiosis, hemolytic anemia, lyme disease, strongyloidiasis, tick-borne coinfection

## Abstract

Babesiosis is a tick-borne infection caused by intraerythrocytic *Babesia* parasites and may result in severe hemolytic anemia and systemic complications. Coinfection with other *Ixodes*-borne pathogens may further complicate diagnosis and management. We report a 45-year-old immunocompetent man working as a landscaper in Connecticut who presented with syncope following two weeks of fever, fatigue, diaphoresis, arthralgias, jaundice, and dark urine. Laboratory evaluation revealed profound hemolytic anemia with a hemoglobin of 6.8 g/dL, elevated lactate dehydrogenase (LDH), undetectable haptoglobin, reticulocytosis, and stage 1 acute kidney injury. Peripheral blood smear demonstrated characteristic Maltese cross formations, with approximately 0.6% parasitemia, and polymerase chain reaction (PCR) testing confirmed *Babesia microti* and *Borrelia burgdorferi* coinfection. Direct antiglobulin testing for immunoglobulin G (IgG) was negative. The patient required intensive care monitoring and transfusion and improved following treatment with atovaquone, azithromycin, and doxycycline. Subsequent outpatient evaluation identified asymptomatic *Strongyloides stercoralis* infection, which was treated with ivermectin. This finding was clinically significant because warm autoimmune hemolytic anemia is a recognized complication of babesiosis and may require corticosteroid therapy, which can precipitate potentially life-threatening *Strongyloides* hyperinfection in patients with occult infection. This case highlights that severe babesiosis may occur despite low-level parasitemia and emphasizes the importance of recognizing coinfections and obtaining a detailed occupational and environmental exposure history, particularly when future immunosuppressive therapy may be considered.

## Introduction

Tick-borne infections are an important cause of morbidity in endemic regions of the United States and may present with overlapping or nonspecific clinical manifestations. Because *Ixodes scapularis* can transmit multiple pathogens, concurrent infections may occur after a single tick exposure. In particular, *Babesia microti* and *Borrelia burgdorferi*, the causative agents of babesiosis and Lyme disease, respectively, share the same primary vector in the northeastern United States. Recognition of coinfection is clinically important because it may complicate the initial presentation and influence diagnostic and therapeutic decision-making [1,2].

Lyme disease, caused by *Borrelia burgdorferi*, is the most commonly reported vector-borne disease in the United States and is particularly prevalent in the Northeast and upper Midwest. The geographic distribution of Lyme disease overlaps substantially with that of babesiosis because both infections are transmitted predominantly by *Ixodes scapularis* ticks [3,4].

Babesiosis is less frequently reported than Lyme disease but can cause severe illness, particularly through intravascular hemolysis and associated organ dysfunction. Because *Babesia microti* and *Borrelia burgdorferi* share the same tick vector and geographic distribution, coinfection may occur in endemic regions. Prior studies have suggested that patients with concurrent babesiosis and Lyme disease may experience a more prolonged or severe clinical course than those with either infection alone [1,2,5].

Lyme disease may present with a broad spectrum of manifestations, ranging from nonspecific constitutional symptoms to dermatologic, neurologic, cardiac, and rheumatologic involvement. Babesiosis often presents with fever, fatigue, chills, and diaphoresis, but clinically significant infection may result in hemolytic anemia, jaundice, and multiorgan dysfunction. Because the early manifestations of these infections may overlap, recognition of coinfection can be challenging, particularly in patients without a recalled tick bite or characteristic rash [2].

*Strongyloides stercoralis* is a soil-transmitted helminth capable of establishing chronic, often asymptomatic infection. Its recognition is particularly important in patients who may require immunosuppressive therapy, as systemic corticosteroids can precipitate potentially fatal *Strongyloides* hyperinfection or disseminated disease. This consideration may become clinically relevant in babesiosis because, although hemolysis is typically related directly to parasitized erythrocytes, warm autoimmune hemolytic anemia has also been described during or following *Babesia* infection and may require corticosteroid therapy [6-8].

Here, we describe an immunocompetent middle-aged man from the northeastern United States who presented with syncope and profound hemolytic anemia requiring intensive care and was diagnosed with *Babesia microti* infection with concurrent *Borrelia burgdorferi* infection. Subsequent evaluation also identified asymptomatic *Strongyloides stercoralis* infection, a clinically relevant finding given the potential need for corticosteroid therapy should post-babesiosis autoimmune hemolytic anemia develop.

## Case presentation

A man in his late 40s with a medical history notable only for gastritis presented to the emergency department after a syncopal episode while walking outdoors. The event was preceded by blurred vision and generalized weakness, followed by loss of consciousness for approximately five minutes. He recovered spontaneously to baseline without postictal confusion or focal neurologic symptoms.

During the preceding two weeks, he had experienced progressive fatigue, intermittent fever with chills, diaphoresis, headache, arthralgias, and unintentional weight loss. He also reported dark urine, dark-colored stools, and progressive jaundice with scleral icterus. He denied abdominal pain, nausea, vomiting, diarrhea, dysuria, or rash. One month before presentation, he had begun working as a landscaper in Connecticut with frequent outdoor and soil exposure, although he did not recall a tick or insect bite. One week before admission, he had received intramuscular benzathine penicillin in the emergency department for presumed streptococcal pharyngitis.

On presentation, the patient was tachycardic with a heart rate of 107 beats/minute and had a blood pressure of 104/66 mmHg. His temperature was 37.8°C (100°F), respiratory rate was 20 breaths/minute, and oxygen saturation was 96% on room air. Physical examination was notable for scleral icterus and mild mucosal jaundice. No focal neurologic deficits or significant cardiopulmonary abnormalities were identified.

Initial laboratory evaluation demonstrated profound normocytic anemia with biochemical evidence of active hemolysis, accompanied by mild hepatic, coagulation, and metabolic abnormalities. Direct antiglobulin testing for immunoglobulin G (IgG) was negative, making IgG-mediated warm autoimmune hemolysis less likely at presentation; complement-specific direct antiglobulin testing was not performed. Serum creatinine peaked at 1.1 from a known baseline of 0.7 mg/dL, which was found to be consistent with Kidney Disease: Improving Global Outcomes (KDIGO) stage 1 acute kidney injury. Selected laboratory findings at presentation are summarized in Table 1.

**Table 1:** Laboratory findings at admission Authors’ compilation from the patient’s medical record ALT: alanine aminotransferase, AST: aspartate aminotransferase, INR: international normalized ratio, LDH: lactate dehydrogenase, MCV: mean corpuscular volume

Laboratory parameter	Patient value	Reference range
Hemoglobin	6.8 g/dL	13.5-17.5 g/dL
Hematocrit	21.1%	41.0%-53.0%
MCV	96.3 fL	83.0-98.0 fL
Platelet count	240 × 10³/µL	150-440 × 10³/µL
Reticulocytes	7.19%	0.5%-2.5%
LDH	1,104 U/L	100-210 U/L
Haptoglobin	<20 mg/dL	30-200 mg/dL
Total bilirubin	1.6 mg/dL	0.2-1.2 mg/dL
Direct bilirubin	0.8 mg/dL	0.10-0.30 mg/dL
AST	79 U/L	10-40 U/L
ALT	69 U/L	10-50 U/L
Albumin	2.6 g/dL	3.3-5.0 g/dL
Sodium	126 mmol/L	135-145 mmol/L
Creatinine	1.1 mg/dL	0.50-1.30 mg/dL
Prothrombin time	17.2 seconds	9.4-12.5 seconds
INR	1.6	0.8-1.1
Fibrinogen	509 mg/dL	200-400 mg/dL

Peripheral blood smear demonstrated intraerythrocytic parasites. Blood polymerase chain reaction (PCR) testing detected *Babesia microti*. Lyme disease testing demonstrated positive total antibody and immunoglobulin M (IgM) immunoblot results, supporting concurrent *Borrelia burgdorferi* infection. The complete microbiologic and serologic evaluation is summarized in Table 2.

**Table 2:** Microbiologic and serologic findings Authors’ compilation from the patient’s medical record PCR: polymerase chain reaction, VISE: variable major protein-like sequence expressed, IgG: immunoglobulin G, IgM: immunoglobulin M

Test	Result	Reference/interpretive criteria	Interpretation
Parasite screen	Present	Absent	Positive parasite screen
*Babesia* species, Giemsa stain	Positive; parasitemia 0.6%	Negative	Microscopic evidence of babesiosis
*Babesia microti* PCR, blood	Detected	Not detected	Molecular confirmation of *B. microti* infection
*Babesia microti* IgG	<1:16	<1:16: negative; 1:16: equivocal; >1:16: positive	Negative
Lyme VISE total IgG/IgM antibody	Positive; 2.290	Laboratory interpretation: positive	Serologic evidence of *B. burgdorferi* infection
*Borrelia burgdorferi* IgM immunoblot	Positive; 23- and 41-kDa bands	≥2 of 23, 39, or 41 kDa bands: positive	Positive IgM immunoblot
*Borrelia burgdorferi* IgG immunoblot	Negative; 66-kDa band only	≥5 of 10 specified bands: positive	Negative IgG immunoblot
*Ehrlichia chaffeensis* IgG	1:128	<1:64: negative; 1:64-1:128: equivocal; ≥1:256: positive	Equivocal
Anaplasma phagocytophilum IgG	<1:80	<1:80: negative	Negative
Direct antiglobulin test, IgG	Negative	Negative	No evidence of IgG-mediated erythrocyte coating at presentation
*Strongyloides* antibody	Positive; 1.87	Laboratory interpretation: positive	Serologic evidence of *Strongyloides* infection

**Figure 1:**
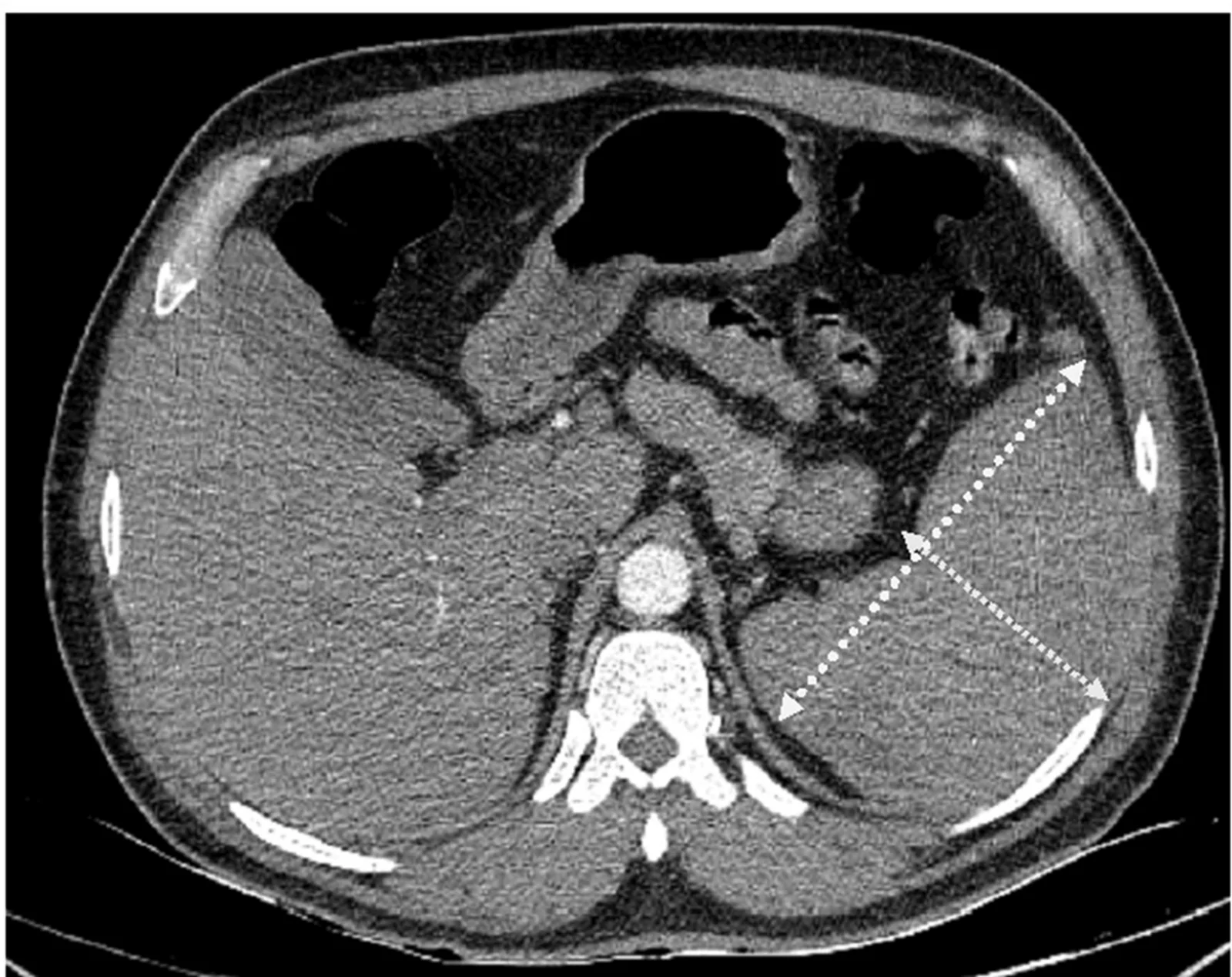
Contrast-enhanced axial computed tomography image of the abdomen demonstrating splenomegaly The dotted measurement lines demonstrate splenic dimensions of approximately 15.1 cm along the long axis and 6.8 cm along the short axis.

**Figure 2:**
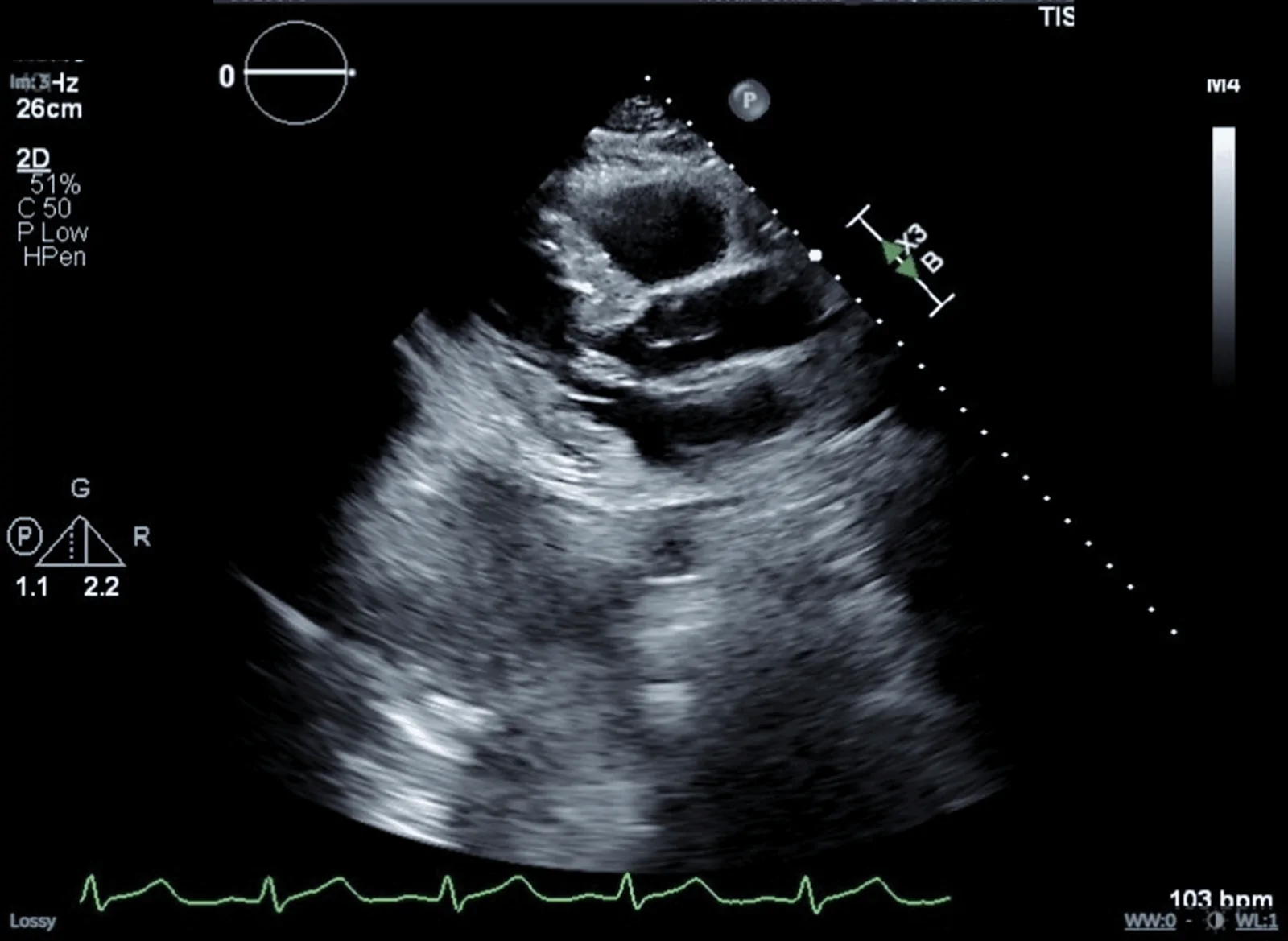
Transthoracic echocardiographic image Echocardiography demonstrated mild left ventricular hypertrophy with preserved left ventricular systolic function (LVEF approximately 60%) and normal wall motion. Right ventricular systolic function was preserved, with no pulmonary hypertension or pericardial effusion. LVEF: left ventricular ejection fraction

Computed tomography of the abdomen demonstrated splenomegaly (Figure 1). Transthoracic echocardiographic findings are shown in Figure 2.

Given the profound symptomatic anemia with syncope, ongoing febrile illness, and evidence of acute organ involvement, the patient was admitted to the medical intensive care unit for close monitoring and treatment. He received one unit of packed red blood cells, after which his hemoglobin stabilized. Antimicrobial therapy with atovaquone 750 mg twice daily and azithromycin 250 mg daily was initiated for babesiosis. Because intermittent fever persisted during the first 48 hours of treatment and concurrent tick-borne infection remained a concern, Infectious Diseases was consulted and doxycycline hyclate 100 mg twice daily was added. Following broadening of antimicrobial therapy, the patient became afebrile and continued to improve clinically.

After clinical stabilization, the patient was transferred to the general medical floor. Hemoglobin values subsequently remained stable and improved from 7.9 g/dL to 8.2 g/dL prior to discharge. He completed a 7-day course of atovaquone and azithromycin for babesiosis and was discharged with a planned 10-day course of doxycycline for Lyme disease coinfection. He was discharged home in stable condition with Infectious Diseases follow-up and repeat assessment of parasitemia. At post-discharge follow-up, additional testing identified asymptomatic *Strongyloides stercoralis* infection, for which ivermectin therapy was initiated.

## Discussion

This case highlights babesiosis presenting with syncope and profound hemolytic anemia despite relatively low-level parasitemia, accompanied by concurrent *Borrelia burgdorferi* infection in an otherwise immunocompetent adult [2]. The subsequent identification of asymptomatic *Strongyloides stercoralis* infection added an important therapeutic consideration because babesiosis has been associated with post-infectious warm autoimmune hemolytic anemia, for which systemic corticosteroids may be required [6]. In patients with occult strongyloidiasis, however, corticosteroid exposure may precipitate potentially life-threatening hyperinfection or disseminated disease [7,8].

Hemolysis in babesiosis is most commonly attributed to destruction of parasitized erythrocytes and associated splenic clearance [6]. In this patient, the marked elevation in lactate dehydrogenase, undetectable haptoglobin, reticulocytosis, hyperbilirubinemia, and profound anemia were consistent with active hemolysis [9]. Direct antiglobulin testing for IgG was negative, making IgG-mediated warm autoimmune hemolytic anemia less likely at the time of presentation. However, complement-specific direct antiglobulin testing was not performed. At that time, autoimmune hemolysis was not strongly suspected, and occult strongyloidiasis had not yet been identified. Warm autoimmune hemolytic anemia has been described during or following babesiosis, including after reduction or clearance of parasitemia. Recognition of this distinction is clinically important because persistent or recurrent hemolysis despite adequate antimicrobial treatment should prompt a more complete evaluation for an immune-mediated process. In retrospect, the subsequent diagnosis of asymptomatic *Strongyloides stercoralis* infection was particularly relevant because the potential need for corticosteroid therapy would have introduced an additional treatment-related risk [6,10].

An additional notable feature of this case was the discordance between the relatively low peripheral parasitemia and the degree of hemolysis and associated clinical complications. Although parasite burden is an important marker of disease severity, clinically significant hemolysis and organ dysfunction may occur even when the percentage of infected erythrocytes is low, which also correlates with current guidelines [2]. Current Infectious Diseases Society of America (IDSA) guidance emphasizes that clinical assessment should incorporate complications such as marked hemolytic anemia and end-organ compromise. Thus, assessment of babesiosis should incorporate the overall clinical picture, including the degree of anemia, evidence of hemolysis, hemodynamic status, and end-organ involvement, rather than relying on parasitemia alone [2]. In this patient, profound symptomatic anemia with syncope, splenomegaly, and KDIGO stage 1 acute kidney injury occurred despite peripheral parasitemia of only approximately 0.6%.

The diagnosis of concurrent *Borrelia burgdorferi* infection also underscores the importance of considering coinfection in patients with compatible epidemiologic exposure. *Babesia microti* and *Borrelia burgdorferi* share the *Ixodes scapularis* vector and overlapping geographic distributions, particularly in the northeastern United States [1,11]. Coinfection may occur even in the absence of a recalled tick bite or characteristic erythema migrans [12]. In this case, recent employment as a landscaper in Connecticut provided a meaningful occupational and geographic exposure history and supported a broad evaluation for vector-borne disease.

The subsequent diagnosis of asymptomatic *Strongyloides stercoralis* infection introduced an important therapeutic consideration. Chronic strongyloidiasis may remain clinically silent for years, but exposure to systemic corticosteroids can precipitate hyperinfection or disseminated strongyloidiasis, particularly in patients with unrecognized infection [7,13]. Because corticosteroids are frequently used in the treatment of clinically significant warm autoimmune hemolytic anemia, identification of occult strongyloidiasis before initiating immunosuppression may be clinically significant [14]. In the present case, corticosteroid therapy was not required because autoimmune hemolysis was not established. Consideration of both geographically endemic pathogens and occupation-related exposures is important when evaluating patients with complex infectious presentations. A comparative summary of the key clinical characteristics, transmission patterns, and treatments of Lyme disease, babesiosis, and strongyloidiasis is provided in Table 3.

**Table 3:** Comparative clinical features and relevance of Lyme disease, babesiosis, and strongyloidiasis in the present case This table summarizes the major microbiologic, epidemiologic, clinical, and therapeutic characteristics of Lyme disease, babesiosis, and strongyloidiasis relevant to the present case. *Borrelia burgdorferi* and *Babesia microti* are transmitted predominantly by *Ixodes scapularis*, whereas *Strongyloides stercoralis* is acquired through exposure to contaminated soil. GI: gastrointestinal, ICU: intensive care unit, LDH: lactate dehydrogenase Source: Compiled by the authors from previously published literature cited in references [2,3,5,7,13]

Feature	Lyme disease (*Borrelia burgdorferi*)	Babesiosis (*Babesia microti*)	Strongyloidiasis (*Strongyloides stercoralis*)
Pathogen type	Spirochete (bacterium)	Intraerythrocytic protozoan parasite	Soil-transmitted helminth
Mode of transmission	*Ixodes* tick bite	*Ixodes* tick bite	Skin contact with contaminated soil
Typical incubation	3-30 days	1-6 weeks	Months to years (chronic infection)
Common symptoms	Fever, fatigue, arthralgia, rash, neurologic or cardiac involvement	Fever, chills, hemolytic anemia, jaundice	Often asymptomatic; GI or pulmonary symptoms
Key laboratory findings	Elevated inflammatory markers	Hemolytic anemia, ↑ LDH, ↓ haptoglobin	Eosinophilia (may be absent)
Severity factors	Delayed treatment, coinfection	Asplenia, immunosuppression, age	Immunosuppression (risk of hyperinfection)
Treatment	Doxycycline	Atovaquone + azithromycin	Ivermectin
Role in this case	Contributed to persistent fever	Primary cause of hemolytic anemia and ICU admission	Incidental but clinically relevant finding

This case emphasizes that peripheral parasitemia alone may not reflect the extent of clinically significant hemolysis in babesiosis. Profound hemolysis and clinically significant complications may occur despite a relatively low parasite burden, requiring integration of laboratory findings, organ involvement, and the overall clinical presentation. In endemic regions, identification of one tick-borne infection should also prompt consideration of concurrent pathogens when supported by the clinical and epidemiologic context. Finally, a detailed exposure history may reveal additional infections that are not responsible for the acute presentation but may substantially influence subsequent therapeutic decisions. In this patient, the later identification of asymptomatic strongyloidiasis illustrates how such findings may become particularly relevant when immunosuppressive therapy is being considered.

## Conclusions

This case demonstrates that the degree of peripheral parasitemia may not fully reflect the extent of hemolysis or associated clinical complications in babesiosis. Profound hemolysis and clinically significant complications may occur despite a relatively low parasite burden, requiring integration of laboratory findings, organ involvement, and the overall clinical presentation. In endemic regions, identification of one tick-borne infection should also prompt consideration of concurrent pathogens when supported by the clinical and epidemiologic context. Finally, a detailed exposure history may reveal additional infections that are not responsible for the acute presentation but may substantially influence subsequent therapeutic decisions. In this patient, the later identification of asymptomatic strongyloidiasis was not relevant to the acute treatment course but would be clinically important if immunosuppressive therapy were subsequently considered.
